# Changes to body mass index, work self-efficacy, health-related quality of life, and work participation in people with obesity after vocational rehabilitation: a prospective observational study

**DOI:** 10.1186/s12889-021-10954-y

**Published:** 2021-05-17

**Authors:** Anita Dyb Linge, Chris Jensen, Petter Laake, Stål Kapstø Bjørkly

**Affiliations:** 1 Institute of Social Sciences, Volda University College, Mailbox 500, 6101 Volda, Norway; 2 Norwegian National Advisory Unit on Occupational Rehabilitation, Haddlandsvegen 20, 3864 Rauland, Norway; 3 Department of Public Health and Nursing, Norwegian University of Science and Technology (NTNU), Håkon Jarls gate 11 and Mauritz Hanssens Gate 2, 7491 Trondheim, Norway; 4 Faculty of Health Sciences and Social Care, Molde University College, Britvegen 2, 6410 Molde, Norway; 5 Oslo Centre for Biostatistics and Epidemiology, Department of Biostatistics, University of Oslo, Sognsvannsveien 9, 0372 Oslo, Norway

**Keywords:** Vocational rehabilitation, Health-related quality of life, Obesity, Return to work self-efficacy, Work ability, Work absence

## Abstract

**Background:**

People on or at risk of sick leave from work due to obesity or obesity-related problems participated in a new vocational rehabilitation (VR). The study aimed to examine the outcome changes in the participants’ health-related quality of life (HRQoL), body mass index (BMI), return to work self-efficacy (RTWSE), work ability scale (WAS) and degree of work participation (DWP) after their participation in the 12-month VR programme. The secondary aim was to examine associations between the outcome changes and HRQoL at 12-month follow-up, measured with the HRQoL 15D instrument (15D).

**Methods:**

This prospective observational study included 95 participants. The one-year multidisciplinary VR programme with an integrated work and lifestyle intervention included 4 weeks of inpatient stay followed-up by 5 meetings. A paired sample t-test was used to examine changes in HRQoL, BMI, RTWSE, WAS, and DWP between baseline and the 12-month follow-up. Multiple linear regression analyses explored associations between changes in HRQoL and the outcome variables.

**Results:**

The participants achieved statistically significant changes in HRQoL (2.57, 95% CI: 1.35 to 3.79), BMI (− 2.33, 95% CI: − 3.10 to − 1.56), RTWSE (15.89, 95% CI: 4.07 to 27.71), WAS (1.51, 95% CI: 0.83 to 2.20) and DWP (18.69, 95% CI: 8.35 to 29.02). At 12 months, a significant association was found between HRQoL and BMI (*B =* − 0.34, 95% CI: − 0.65 to − 0.04), RTWSE (*B =* 0.02, 95% CI: 0.004 to 0.04), WAS (*B =* 0.91, 95% CI: 0.55 to 1.28), DWP (*B =* − 0.02, 95% CI: − 0.04 to 0.001) and work absence (*B =* − 0.01, 95% CI: − 0.02 to − 0.002). The regression model explained 71.8% of the HRQoL variance.

**Conclusion:**

The results indicated positive changes in HRQoL, BMI, RTWSE, WAS and DWP from baseline to the 12-month follow-up. Factors associated with HRQoL at the 12-month follow-up were decreased BMI, increased RTWSE, improved WAS and reduced work absence. Future studies examining VR programmes with lifestyle interventions for people with obesity are recommended.

**Trial registration:**

Norwegian Regional Committee for Medical and Health Research Ethics (REC) 2017/573, Clinical Trials NCT03286374, registered 18. September 2017.

https://clinicaltrials.gov/ct2/results?cond=Obesity&term=Anita+Dyb+Linge&cntry=NO&state=&city=&dist=

**Supplementary Information:**

The online version contains supplementary material available at 10.1186/s12889-021-10954-y.

## Background

Obesity is increasing rapidly and presents a health challenge in most parts of the world. People with obesity may have a high quality of life; however, obesity (body mass index [BMI] ≥ 30 kg/m^2^) is generally associated with severe health complications, functional impairment and lower self-esteem [1]. Many people with obesity are subjected to humiliation, stigmatisation, discrimination and bullying, and for some of them, such discrimination is a daily experience in many arenas of their social life [1–3], including the workplace [4]. Furthermore, people with obesity (of all ages) may experience the loss of working capacity and may struggle to enter and remain in the workforce [5, 6]. This can contribute to adverse financial and social consequences, as well as feelings of isolation and depression [1, 7].

The many negative consequences of obesity contribute to a poorer health-related quality of life (HRQoL) when compared to the non-obese population [8–10]. HRQoL, as defined by the World Health Organization (WHO), indicates the individual’s perspective (related to health status, values, levels of satisfaction and general well-being) of a specific health condition [11]. HRQoL includes *“the physical, psychological and social domains of health which are influenced by a patient’s experiences, beliefs and expectations of their condition and treatment”* [11].

The Norwegian government promotes an active labour and welfare policy that aims for participation in working life by as many people as possible [12]. Therefore, to manage problems related to health and functional capacity and to adjust contextual factors, the government provides vocational rehabilitation (VR) to help individuals who struggle to remain in the workforce. VR aims not to cure illness but to promote work participation despite health complaints and sickness. Often, the outcome and final goal of VR is return to work (RTW). Previous research has indicated that people attending VR programmes may enhance work ability, reduce sick leave, achieve earlier RTW following sick leave and reduce work disability [13, 14].

Work participation requires sufficient work ability, which is a key concept to address when rehabilitating working-age people [7, 15]. In the International Classification of Functioning, Disability and Health (ICF) framework, *work ability* describes the functional ability to perform work, as well as the interaction between individuals’ physical and mental factors, along with various social and environmental factors [15]. Reduced work ability is a significant factor in reduced work participation for people with obesity in different occupational groups [16].

However, VR addresses only a small part of the complex picture of rehabilitation [7]. People who participate in VR programmes tend to have more complex needs due to health problems, length of sick leave and circumstances at home or work. Contact with appropriate agencies, such as the workplace and Norwegian Labour and Welfare Administration (NAV), may provide sustained work capacity and job satisfaction among individuals and increase the prerequisites for RTW.

In Norway, traditional VR programmes tend to last no more than 4 weeks, and only occasionally up to 12 weeks [12]. This short duration contrasts sharply with the time people with obesity need to make lifestyle changes, which preferably would be more than 6 months [17].

Before 2015, no VR programme in Norway focused on both work and lifestyle intervention. Therefore, a new, temporary, multidisciplinary rehabilitation programme for people with obesity that focused on enhanced work self-efficacy and lifestyle change was established in a specialised rehabilitation centre. The new VR programme differed from traditional VR programmes through its inclusion of dietary and cognitive behavioural interventions, as well as its length. The programme was built on the WHO ICF framework [13], combined with the Sherbrook workplace model and intervention [18]. It consists of practical and theoretical intervention components that are applied in groups and individually. Furthermore, the inpatient VR programme was established with several follow-ups over an entire year.

The integrated treatment approach, which focuses on work activity, diet and physical activity, uses cognitive approaches to develop coping strategies and self-efficacy skills, aiming to contribute to body weight loss and RTW [19]. The VR programme emphasises self-efficacy as an essential element of human motivation and behaviour that affects how individuals embrace and cope with life [20]. According to Bandura, higher self-efficacy is an important determinant of behaviour change [20–23]. Self-efficacy, which describes personal motivation and the development of coping strategies, may be a useful concept for understanding the self-management aspects of RTW [7]. Therefore, a specific measurement of RTW self-efficacy (RTWSE) may be useful for evaluating VR interventions and capturing elements of both personal motivation and situational barriers [24].

The gap between published research results from VR programmes with and without lifestyle interventions is large. Several books [25, 26] and journal articles [27, 28] have addressed conceptual models of work disability and RTW [28], but little to no focus has been given to obesity and lifestyle change [29]. Only a few studies have focused on the association between multifactorial lifestyle risk and work ability [30]. For example, a Polish study found that work ability was strongly associated with lifestyle for both men and women [18]. In a Norwegian study, low work ability was more likely to be observed in individuals with unhealthy diets, inactivity and,- obesity, as well as those who were former or current smokers [30]. To the authors knowledge, no other published research has primarily focused on treatment outcomes from participation in VR with integrated work and lifestyle intervention for people on or at risk of sick leave from work due to obesity or obesity-related problems.

Measuring HRQoL may provide information about the burden of obesity beyond the treatment goals and describe the effects of diseases and treatment from the participants’ view [10, 31]. Therefore, the primary aim of the present study was to examine the outcome changes in HRQoL, BMI, RTWSE, work ability (as measured with the work ability scale [WAS]), and work participation (measured as the degree of work participation [DWP]) from baseline to 12-months follow-up for participants with obesity in a VR programme with a lifestyle intervention. The secondary aim was to examine associations between the outcome changes and HRQoL at 12-month follow-up. Many studies on obesity and body weight loss have included HRQoL, however, few have explored the mediators affecting HRQoL [32]. Four previous reviews strongly recommended exploring mediators to understand changes in HRQoL [33–36].

## Methods

### Participants

The study’s participants were recruited from a publicly-funded VR programme with a lifestyle intervention at Muritunet Rehabilitation Centre in Western Norway. The individuals were referred to the programme by general practitioners according to their right to admission to Norwegian specialist health services [12]. In total, 190 eligible people (divided into 18 groups) stayed at the rehabilitation centre between April 2015 and December 2017. Of these, 95 agreed to participate in the study. Due to the nature of the study intervention, blinding the participants was not possible. However, the employees of the VR programme were blinded to which individuals were participants in the study.

Inclusion criteria were as follows: participants on or at risk of sick leave from work due to obesity or obesity-related problems, had a BMI of more than 30 kg/m^2,^ were 18 to 67 years old, and had a realistic opportunity to work part- or full-time. The exclusion criteria included substance and alcohol abuse, unstable medical conditions that prevented physical activity, pregnancy, severe mental illness, disability pension, and disabilities requiring permanently modified work.

### Study design and setting

**Fig. 1:**
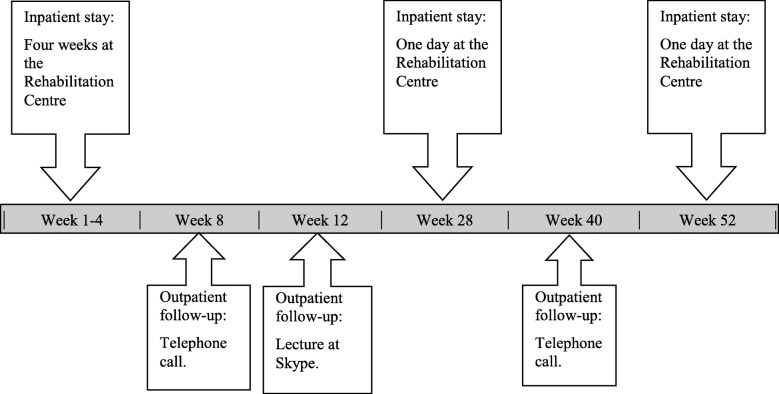
A schematic overview above in-and outpatient stay of the vocational rehabilitation programme

The prospective observational study examined the outcomes of a one-year VR programme. The programme commenced with 4 weeks of inpatient stay at the rehabilitation centre, followed by 5 follow-up meetings at 8, 16, 28, 40 and 52 weeks after baseline (Fig. 1). The multidisciplinary team engaged in the rehabilitation programme comprised a labour consultant, health care professionals and a sports educator; they all had complementary roles and collaborated to assess and treat the participants. Each participant developed a plan with goals for work activity, diet, physical activity, and coping strategies for the rehabilitation period.

During the 4-week inpatient stay, the participants had, on average, 17 h of individual consultation with a labour consultant, medical doctor, dieticians, physiotherapist, psychiatrist or nurse, all of whom were educated in Motivational Interviewing [37]. The participants also took part in 48 h of group activity and lectures, distributed as follows: 14 h of cognitive behaviour theory and practice, 20 h of physical activity, 9.5 h of dietary education, 2.5 h of work-related education and 2 h of lectures about obesity. After the inpatient stay, the participants received an additional 4 h of individual consulting, 6 h of cognitive behaviour theory and practice, 2 h of food education and 4 h of physical activity.

#### Variables

The study used baseline data that was collected prior to the intervention and data that was collected at the last, 12-month follow-up. At baseline, the participants answered questionnaires and took body composition tests on either the first or second day of the visit. After 12 months, the participants brought completed forms to the follow-up and took body composition tests at the rehabilitation centre. Participants who did not attend the 12-month follow-up were encouraged to return completed forms by mail with an updated body weight. Non-responders were sent one reminder.

### Health-related quality of life measure

The outcomes were measured by the 15D instrument (15-dimensional) published by Sintonen [38]. The 15D consists of many different health states and therefore embraces diverse arenas important for people with obesity [38, 39]. It is a generic, comprehensive, self-administered instrument for measuring HRQoL among adults (aged 16+ years). The 15D covers most of the *“domains of health”* emphasised in the WHO ICF [13], it measures mobility, vision, hearing, breathing, sleeping, eating, speech, excretion, usual activities, mental function, discomfort, symptoms, depression, distress, vitality and sexual activity. The 15D describes health status at five ordinal levels for each dimension (1 = no problem with any aspect, 5 = deceased). The score was reversed before the analyses so that a higher score indicated better HRQoL. To obtain adequate score variation in the small sample, the total sum score of all 15 question were used (range 1 to 75); previous studies on individuals with musculoskeletal, cardiovascular or psychosomatic disorders and on obese patients, have confirmed better responsiveness to change when assessed by the total scores [40, 41].

The 15D is comparable with existing, commonly used profile and single index scoring instruments in term of reliability, validity, sensitivity, discriminatory power, and response to change [38, 40, 42].

A minimally important difference (MID) is used to provide a measure of the smallest change in the 15D instrument that the participants would identify as important for their HRQoL. A MID should be approximately half a SD [43].

### Body mass index

Bodyweight (kg) was measured with a Tanita MC-780 U Multi Frequency Segmental Body Composition Analyzer. The participants’ weight was measured with light clothing and without shoes in the morning before breakfast. Each person’s height (cm) was measured in a standing position without shoes using a stadiometer. Height was added to the Tanita MC-780 U, and BMI was calculated as weight (kg) divided by height (m) squared and was reported in kg/m^2^. The following WHO BMI reference values for adults were used: underweight (< 18.5), normal weight (18.5 to 24.9), overweight (25.0 to 29.9), obesity class I (30.0 to 34.9), obesity class II (35.0–39.9), and obesity class III (above 40) [44]. Participants who were unable to attend the 12-month follow-up reported their self-monitored weight.

### Return to work self-efficacy

RTWSE is a reliable and valid measurement for assessing working adults with musculoskeletal disorders in terms of their confidence to meet job demands, modify job tasks and communicate needs to co-workers and supervisors [24, 45]. RTWSE was expected to be a reliable tool for obese individuals with somatic symptoms, as one-third of the present study’s participants reported musculoskeletal problems. To determine RTWSE, 19 questions with a score ranging from 1 (*“not sure at all”*) to 10 (*“very sure”*) were used to determine RTWSE. The total score (range, 0 to 190) was used for the analyses. RTWSE scoring by Shaw et al. [24] was followed to interpret changes in self-efficacy, as follows: < 5, low self-efficacy; 5 to 7.5, medium self-efficacy; and > 7.5, high self-efficacy. Higher values indicated a more positive self-reported RTWSE [24].

### Work ability score (WAS)

The WAS involved a self-assessment of perceived mental and physical capacity and work demands [30]. Self-rated work ability was assessed using a single-item question to determine the WAS; this question was published by Gould et al. [46] as part of the full work ability index (WAI) [47]. Previous studies have demonstrated a strong association between WAS and the complete WAI [47, 48]; change in the single-item question predicts the future degree of sick leave, HRQoL, vitality, neck pain, self-rated general physical and mental health, lifestyle and behaviours and current stress [16, 47]. The question used by this study to measure WAS (*“current work ability compared with your lifetime best”*) used a scale of 0 to 10 (0 = *“completely unable to work”* and 10 = *“work ability at its best”*). The following measurement classification from Gould et al. [46] was used: poor (0 to 5), moderate (6 to 7), good (8 to 9) and excellent (10).

### Degree of work participation

DWP ranged from 0 to 100 (in per cent) and is the percentage of a full-time position. The first measurement (DWP baseline) was obtained 5 days before the patients enrolled in the programme to avoid counting sick leave used to participate in the program. The second measurement was obtained at the 12-month follow-up. If work participation was not continuous for at least 4 weeks after 12 months follow-up due to vacation and temporary absence, DWP was assigned a value of 0%. For those with 4 weeks of continuous work participation after 12-month follow-up, DWP was based on the degree of their current work participation. DWP data was obtained from by NAV.

### Work absence

Work absence was measured as the number of days absent from work from baseline to 12-month follow-up, as obtained from the NAV. The number of days was adjusted according to reimbursement of part- or full-time benefits, normal working hours for each individual and adjustment for the 2016 leap year. Changes in lifestyle and work self-efficacy are associated with absence days and HRQoL [6, 49, 50], so measuring work absence during the rehabilitation period was reasonable.

### Sociodemographics

Age, gender, ethnicity, and education level were obtained from the patients’ records at the rehabilitation clinic. Age referred to the age of the participants at the start of the intervention. Education level was divided into three categories: (1) elementary school, < 10 years; (2) high school, < 14 years; and (3) college/university education, > 14 years.

Social benefits, work participation (full- or part-time) and sick leave diagnoses were obtained from the NAV. The diagnoses were coded and grouped according to the International Classification of Primary Care (ICPC-2).

### Statistical analysis

Data were analysed using SPSS, v.27 (Armonk, NY: IBM Corp.).

The characterise descriptive statistics were produced with the mean, standard deviation (SD) and range for continuous variables and with numbers and valid percentages for categorical variables. Assumption of normality was tested with a P-P plot (Additional file 1). Paired sample t-test were used to analyse HRQoL, BMI, RTWSE, WAS, and DWP for differences between baseline and the 12-month follow-up.

Simple and intermediate multiple regression analyses formed the basis for testing variables in a final multiple linear regression analysis. The explanatory variables were included and excluded according to F-tests for changes in R^2^. Simple regression analyses were used to produce unadjusted regression effects, and the final regression analysis produced the adjusted regression effect for HRQoL at the 12-months follow-up relative to the change in BMI, RTWSE, WAS, DWP and work absence. These variables were all adjusted for age, gender, sick leave diagnosis, education level, and HRQoL at baseline. All regression effects were presented as unstandardized coefficients.

To detect any potential correlation between the explanatory variables in the final multiple regression analysis, the variance inflation factor (VIF) was examined [51]. Furthermore, P-P-plots were used to examine how closely the data sets agreed and to evaluate the plot distribution’s skewness [52].

The sample size calculation was based on the results from a group that had previously participated in a similar occupational rehabilitation intervention at Muritunet Rehabilitation Centre. For HRQoL an effect size of 0.4 was assumed. Therefore, a sample size of 55 was needed to obtain a power of 80% with a significance level of 5%.

## Results

For this study, 95 Caucasians with a mean age of 47 years (range 19 to 64 years) consented to participate. Of these, 46.3% were female. Scores for HRQoL, BMI, RTWSE, WAS, DWP, and work absence were calculated for 68 (71.6%), 65 (68.4%), 62 (65.3%), 68 (71.6%), 95 (100%) and 95 (100%) respondents, respectively. Due to weight loss while waiting to enter the program, one patient had a BMI of 29. The most prevalent sick leave diagnoses were related to musculoskeletal diseases, as well as endocrine, nutritional and metabolic diseases. Among “other diagnoses,” almost 70% of the group presented with mental health symptoms, according to the NAV. During the rehabilitation period (from baseline to the 12-month follow-up), the participants had, on average, 152 days of work absence. Of the participants, 83% had continuous absence at the start of the rehabilitation period, while only 16% had periodic absence throughout the rehabilitation period. One patient did not record work absence during the rehabilitation period; this participant attended the rehabilitation program between work shifts.

After 12-month, 27 participants (12 females and 15 males) were lost to follow-up. Of these, 19 participants did not report any reason, 4 could not come due to work, 2 transitioned to disability pension, 1 reported as sick, and 1 patient moved to another part of Norway. The 27 missing participants contributed only minimal differences to the background, outcome and explanatory variables. The individuals who did not participate in this study had lower values in reported body weight, age and education level, and they included more females (Table 1).

**Table 1:** Characteristics of the participants, Baseline

	Follow-up *N* = 68		Lost to follow-up *N* = 27 ^d^		Not included *N* = 95 ^e^	
**Outcome variable**						
HRQoL, mean (SD ^a^, range^b)^	64.4	(5.43, 53–75)	64	(5.9, 50–75)		
**Explanatory variables**						
BMI, mean (SD, range)						
Body mass index, kg/m^2^	38.8	(4.2, 29–48.5)	40.5	(5.5, 30.2–57.8)	38.5	(5.5, 27.3–57.3)
Weight, kg	121.1	(18.9, 74.9–170.4)	123.5	(20.5, 74.9–196)	116.3	(20.6, 68.1–165.2)
High, cm	176.4	(9.9, 521–200)	174.9	(10.8, 152–196)	173.2	(8.6154–197.5)
RTWSE, mean (SD, range) (*n* = 94)	124.2	(38.1, 29–183)	128.2	(40, 41–190)		
WAS, mean (SD, range)	5.7	(2.7, 0–10)	5.4	(3, 0–10)		
DWP, mean (SD, range)	47.23	(46.68, 0–100)	41.85	(48.68, 0–100)		
Days of work absence, mean (SD, range) ^c^	152	(124, 0–365)	156	(131, 20–365)		
**Background variables**						
Sociodemographic status, n (%)						
Age, mean (SD, range)	47.6	(9.5, 23–63)	45.8	(11.5, 19–64)	46	(9.9, 20–66)
Gender, n (%)						
Males	36	(53)	15	(55.6)	43	(45.3)
Females	32	(47)	12	(44.4)	52	(54.7)
Sick leave diagnoses, n (%)						
Musculoskeletal system	23	(35.9)	11	(40.7)		
Endocrine, nutritional, and metabolic diseases	17	(26.5)	12	(44.4)		
All other reported diagnosis	24	(37.5)	4	(14.8)		
Education level, n (%)						
Collage/university education (> 14 Years)	31	(45.6)	8	(29.6)	31	(33)
High school (< 13 Years)	29	(42.6)	16	(59.3)	43	(45.7)
Elementary school (< 10 Years)	8	(11.8)	3	(11.1)	17	(18.1)
Not finished elementary school					3	(3.2)

^a^ Standard deviation: SD

^b^ Range; Minimum and maximum value

^c^ Work absence, measured as number of days from baseline to 12-month follow-up. Numbers of days are adjusted for leap year in 2016

^d^ Baselinevalues for 27 missing participants at 12 -month follow-up

^e^ Participants who declined to participate in the study

At baseline, 11 participants were not in receipt of social benefits from the NAV. They participated in the rehabilitation programme between work shifts. Therefore, it was presumed that they would report higher confidence in their current ability to resume normal job responsibilities. Furthermore, at 12-months, another group (*n* = 11 participants) did not appear but reported their self-observed body weight. The findings indicated that the bias by these two groups was minimal.

The results of the primary analyses of the changes from baseline to the 12-month follow-up, showed a statistically significant change in HRQoL, BMI, RTWSE, WAS, and DWP during the rehabilitation programme (Table 2). The changes in HRQoL from baseline to 12-month follow-up was 2.57, which was approximately half an SD at baseline. Thus, a minimal important difference (MID) for clinically significant change was obtained [43].

**Table 2:** Comparison of HRQoL, BMI, RTWSE, WAS and DWP from baseline to 12-months follow-up

	Measure	Mean changes from BL to 12-months	95% CI	*p* values
(*n* = 68)	HRQoL	2.57	1.35: 3.79	**< 0.001**
(*n* = 65)	BMI	−2.33	− 3.10: − 1.56	**< 0.001**
(*n* = 68)	WAS	1.51	0.83: 2.20	**< 0.001**
(*n* = 95)	DWP	18.69	8.35: 29.02	**0.001**

A paired sample t-test was conducted for each of the outcome variables

Confidence interval: CI

Statistically significant variables (≤ 0.05) are marked with bold

BMI decreased from 38.8 kg/m^2^ to 36.4 kg/m^2^, a reduction of 6% from baseline to 12-month follow-up, still recorded in the category obesity II (BMI 35–39.6). The RTWSE score increased by 13% from baseline (6.49) to the 12-months follow-up (7.32), and the participants scored in the upper level of the moderate belief category concerning RTWSE.

From baseline to the 12-month follow-up, the number of participants with a poor WAS score (0 to 5 points) decreased from 32 to 17. The number of participants with a good score (6 to 7) decreased from 13 to 12. Those with a moderate score (8 to 9) increased from 20 to 25, while participants with an excellent score (10) increased from 3 to 14. In average, for all participants, the WAS increased from 5.7 to 7.2, moving from the poor category to the moderate category during the rehabilitation period.

From baseline to the 12-month follow-up, the work participation of the participants increased from 45.7 to 64.4%. DWP increased from 45.7 to 64.4%. At baseline, 65% received social benefits from the NAV, and 35% of participants worked either part or full time. After the 12-month follow-up, 73% of the participants worked part- or full time, an increase of 52% from baseline.

The secondary aim was to examine associations between the outcome changes and HRQoL at 12-months follow-up. The unadjusted analysis estimated the effect of explanatory variables (BMI, RTWSE, WAS, DWP, and work absence in the follow-up period), and background variables (age, gender, sick leave diagnosis, education level, and HRQoL at baseline) on HRQoL at the 12-month follow-up. Those diagnosed with diseases of the musculoskeletal system and “all others” reported lower HRQoL than participants with diseases of the endocrine, metabolic or nutritional system. Due to the F-test for changes in R^2^, DWP was included in the final analyses event though a *p* = 0.242. Educational level was not statistically significant and was excluded from further analyses.

A final multiple linear regression analysis was performed to analyse the association between HRQoL at the 12-month follow-up with changes in the explanatory variables BMI, RTWSE, WAS, DWP and work absence in the 12- month follow-up period. These variables were adjusted for HRQoL baseline and gender (Table 3). The regression model explained 71.8% of the variation in HRQoL at the 12-month follow-up (F [7, 48] = 17.48, *p* < 0.001). Without HRQoL at baseline, this model explained 41.6% of the variance in HRQoL at 12-months follow-up (F [6, 49] = 5.82, *p* < 0.001). HRQoL at the 12-month follow-up was statistically significantly associated with a decrease in BMI, increased RTWSE and WAS and fewer days of work absence.

**Table 3:** Associations between HRQoL at 12-months follow-up and the other outcome variables; BMI, RTWSE, WAS, DWP and work absence, adjusted for the background variables. Unadjusted and adjusted multiple regression analyses

	Unadjusted analysis:			Final analysis:		
	*B* ^a^	95% CI	*p* values	*B*	95% CI	*p* values
Age	0.12	−0.02: 0.27	**0.100**			
Gender						
Male	0			0		
Females	−0.38	−3.23: 2.47	0.792	2.07	0.031:4.10	**0.047**
Sick leave diagnosis			**0.015**			
MS ^b^	0					
EMD ^c^	4.89	1.45: 8.32	0.006			
All other ^d^	3.2	0.09: 6.31	0.044			
Educational level			0.267			
Collage/university (< 14 years)	2.26	−0.74: 5.25	0.137			
High school (> 13 years)	0					
Elementary school (> 10 years)	2.59	−2.04: 7.22	0.268			
HRQoL (baseline)	0.65	0.44: 0.86	**< 0.001**	0.73	0.53: 0.94	**< 0.001**
BMI ^e^	−0.59	−0.10: −0.05	**0.031**	−0.34	− 0.65:-0.04	**0.029**
RTWSE ^e^	0.04	0.01: 0.07	**0.021**	0.02	0.004: 0.04	**0.023**
WAS ^e^	0.86	0.43: 1.34	**< 0.001**	0.91	0.55: 1.28	**< 0.001**
DWP ^e^	0.017	0.01:0.05	**0.242**	−0.019	−0.04:0.001	0.060
Work absence ^f^	−0.02	−0.03: − 0.01	**0.001**	−0.01	− 0.02: − 0.002	**0.020**

Univariate analyses. Variables were included and excluded according to F-tests for changes in R2 marked with bold and further included in the final analyses

Multiple regression analysis: Final analyses are controlled for the effect of background variables and HRQoL baseline

^a^ Unstandardized regression coefficient (B)

^b^ Musculoskeletal diagnosis

^c^ Endocrine, metabolic, and nutritional diagnosis

^d^ All other reported diagnosis

^e^ Value of change from baseline to 12-months

^f^ Work absence, measured as the number of days from baseline to 12-month follow-up

In the final analyses; statistically significant *p* values (≤ *0*.05) marked in bold

*R*^2^ Final analysis = 71,8 F = 17,48

No consequential multicollinearity was found between the explanatory variables in the VIF estimates of the final model. The Mahal and Cooks distance indicated no extreme cases that affected the model. The P-P plot between the expected and the observed cumulative distributions was considered acceptable (Additional file 1).

## Discussion

The present study examined the participants’ changes in HRQoL, BMI, RTWSE, WAS and DWP from baseline to the 12-month follow-up after participation in a VR programme with specific components to address lifestyle change. The participants achieved a statistically significant increase in HRQoL and the treatment goals concerning BMI reduction, work self-efficacy and work participation. A change in HRQoL of approximately half an SD indicated a clinically significant change that could help the participants feel more satisfied and successful with the treatment outcome and their lives. Furthermore, the participants’ changes showed a 6% BMI loss, which could contribute to a reduced risk of cardiovascular diseases and help to prevent early mortality [10]. The participants also increased their RTWSE to moderate belief about their current ability to resume and handle normal job responsibilities. Additionally, the participants’ WAS score increased during the VR programme, from poor to moderate, as did DWP which could contribute to individual well-being and reduced social security costs to society [7]. These changes indicated progress in the participants’ RTW progress, HRQoL, vitality, self-rated general physical and mental health, lifestyle and behaviours and current stress [15, 16, 47]. Furthermore, the participants’ self-efficacy beliefs about changing their behaviour seemed to be strengthened during the VR programme.

The paper’s second aim was to examine associations between the outcome changes and HRQoL at 12-months follow-up, measured with the 15D. A statistically significant association was found between HRQoL at the 12-month follow-up and changes in BMI, RTWSE, WAS and days of work absence. The final regression model fit the data well, with 71.8% explained variance, which provided a good indication of factors that contribute to variation in HRQoL.

The major difference between traditional VR programmes and the one examined by this study was the focus on dietary- and cognitive behavioural intervention, as well as the programme’s length. The findings in this study and for this new programme were the participants’ change in BMI and work participation, which were also associated with HRQoL.

### Comparison with other studies

The findings of the present study were comparable with previous research that indicated that people attending VR programmes may increase work ability and reduce sick leave and work disability [13, 14, 22].

The results of the present study showed that focus on lifestyle intervention over time resulted in BMI reduction; this was also comparable to the findings of other studies on lifestyle interventions [17]. Previous published studies on people with obesity found significant variability in HRQoL after weight loss [32]. However, the findings of the present study supported other published studies that found that weight loss is related to HRQoL [10, 32, 39]. The results also aligned with those other studies that found improved HRQoL scores correspond with increased positive self-efficacy-expectations and work participation, indicating that employment may positively contribute to self-esteem [20, 31, 53].

Furthermore, the participants’ WAS scores were comparable to those found in other studies that showed an association between levels of obesity and WAS [16, 54, 55]. Additionally, the process of returning to work following a long-term sick leave can be challenging to individuals, both physically and mentally. Participants with the highest number of absence days scored the lowest on HRQoL, and these findings were comparable with those of other studies [32, 50, 56].

Previous studies have focused on obesity and obesity treatment or employment and vocational rehabilitation. To the authors knowledge, no studies have considered VR with a lifestyle intervention lasting 1 year. Based on this research, emphasising and exploring further factors associated with HRQoL for people with obesity are highly recommended.

### Limitations

This study had several limitations. First, since it was a small-scale study, more extensive studies are needed to address participants’ changes during VR programmes with lifestyle interventions, as well as the factors contributing to HRQoL for people on or at risk of sick leave from work due to obesity or obesity-related problems. Second, although the differences between the sample and the non-participants were controlled for, the study failed to compare some important variables due to confidentiality agreements. Third, the study only included individuals who participated in rehabilitation. Therefore, the factors associated with HRQoL may have been influenced by the participants’ involvement in a clinical research study during rehabilitation. Whether the results were representative of a broader population of obese individuals cannot be determined. Fourth, self-reported body weight should be avoided as far as possible because of a potential bias of underestimation [57]. However, no significant body weight was observed between the 11 participants who self-reported their body weight at 12-month and the participants who did not self-report. Finally, the results may not be valid for non-Caucasian people.

### Strengths

The paper’s main strength was its focus on participants who were on or at risk of sick leave from work due to obesity or obesity-related problems, and the VR programme integrated work and lifestyle intervention. Moreover, due to the prospective observational design, the VR programme and the study’s findings may be generalised more easily than if specially developed interventions and a randomized controlled trial design had been used, for example. Finally, different data sources provide a wide range of explanations on factors affecting HRQoL.

## Conclusion

For the first aim, which were related to changes from baseline to the 12-month follow-up, statistically significant positive changes in HRQoL, BMI, RTWSE, WAS and DWP were found after participation in the VR programme. Regarding the second aim, a positive change in HRQoL was significantly associated with BMI loss, improved RTWSE and WAS and a decrease in absence days after participation in the VR programme. The results indicated that people on or at risk of sick leave from work due to obesity or obesity-related problems may need lifestyle changes and return to work self-efficacy to strengthen their HRQoL. Therefore, use of the 15D instrument was appropriate for estimating the outcome of VR programmes and interventions. We hope the findings of this study can inform and inspire future research on VR with a lifestyle intervention for people on or at risk of sick leave from work due to obesity or obesity-related problems.

## Supplementary Information

**Additional file 1.** P-P-plotts. Assumption of normality was tested with P-P plots and were used to examine how closely the data sets agreed and to evaluate the plot distribution’s skewness.

## Data Availability

The dataset used and analysed in this article is available from the corresponding author on reasonable request.

## Abbreviations

HRQoL: Health-Related Quality of Life
BMI: Body Mass Index
RTWSE: Return to Work Self-Efficacy
WAS: Work Ability Score
DWP: Degree of Work Participation
Cm: Centimetres
Kg: Bodyweight
NAV: Norwegian Labour and Welfare Administration
WHO: World Health Organization
ICPC-2: International Classification of Primary Care
VIF: Variance Inflation Factor
