# Supplementary material for: Changes to body mass index, work self-efficacy, health-related quality of life, and work participation in people with obesity after vocational rehabilitation: a prospective observational study
Source: BMC Public Health. 2021 May 17;21:936. doi: 10.1186/s12889-021-10954-y (PMC8130265; doi:10.1186/s12889-021-10954-y)
Supplement: Supplementary file 1 — Additional file 1. P-P-plotts. Assumption of normality was tested with P-P plots and were used to examine how closely the data sets agreed and to evaluate the plot distribution’s skewness. [file 12889_2021_10954_MOESM1_ESM.doc]

Additional file 1 

P-P-plots: Changes between baseline to 12-month follow-up

BMI


RTWSE

HRQoL


WAS


DWP


P-P-plotts
